# PD-L1 immunohistochemical assays for assessment of therapeutic strategies involving immune checkpoint inhibitors in non-small cell lung cancer: a comparative study

**DOI:** 10.18632/oncotarget.21567

**Published:** 2017-10-06

**Authors:** Hyojin Kim, Hyun Jung Kwon, Soo Young Park, Eunhyang Park, Jin-Haeng Chung

**Affiliations:** ^1^ Department of Pathology, Seoul National University Bundang Hospital, Seongnam, Republic of Korea; ^2^ Department of Pathology, Seoul National University College of Medicine, Seoul, Republic of Korea

**Keywords:** programmed cell death-ligand 1, immunotherapy, immunohistochemistry, biopsy, non-small cell lung cancer

## Abstract

Although immune checkpoints inhibitors have exhibited promising activity in clinical trials in non-small cell lung cancer (NSCLC) patients, the current programmed cell death-ligand 1 (PD-L1) assays are inconsistent in terms of the staining analysis and scoring system used. To verify the interchangeability of the available PD-L1 assays, we performed immunohistochemistry using three antibody clones used in clinical trials (22C3, SP263, and SP142) and the E1L3N clone as a laboratory developed test for 97 resected NSCLC specimens. Matched tissue microarray specimens were also stained. Staining with 22C3 yielded a greater proportion of stained tumor cells, whereas SP142 staining consistently labelled fewer tumor cells. However, when various cut-off criteria were applied, the positivity rates for PD-L1 were similar, with high concordance, under assay-specific cut-offs. Moreover, seven cases of discordant PD-L1 expression between the resected specimen and matched tissue microarray specimens were observed. In conclusion, despite of inter-assay variability of the PD-L1 status in NSCLC, the positivity rate appears to be similar under assay-specific criteria. Hence, an appropriate clinically defined algorithm or cut-off should be separately applied for each assay. Moreover, multiple biopsy specimens from different tumor areas should be obtained to reduce false results due to intratumoral heterogeneity in PD-L1 expression.

## INTRODUCTION

Programmed cell death 1 (PD-1)/PD-1 ligand 1 (PD-L1) checkpoint inhibitors for heavily pre-treated patients with advanced non-small cell lung cancer (NSCLC) represent major advances in immunotherapy [1, 2]. Recent data have led to the approval of three PD-1/PD-L1 inhibitors, including nivolumab, pembrolizumab, and atezolizumab, for the treatment of advanced NSCLC after first-line therapy [3–7]. However, their overall response rates in unselected populations are low, emphasizing the need for predictive biomarkers to identify the most suitable patients.

Recently approved tests for anti-PD-1/PD-L1 therapy in NSCLC include the assessment of PD-L1 expression using immunohistochemistry (IHC) as a companion diagnostic test (22C3 for pembrolizumab) [5, 7] and 2 complementary diagnostic tests (28-8 for nivolumab and SP142 for atezolizumab) [3, 4, 8]. Another PD-L1 assay (SP263) is currently being tested in clinical trials [9, 10]. Further, laboratories and research institutions also use laboratory-developed tests (LDTs), most notably using the E1L3N clone [11]. However, the PD-L1 expression status and its predictive and prognostic values differ considerably with different antibody clones, platforms, and interpretation criteria [12–15].

Although companion/complementary PD-L1 assays are developed using a ‘one drug–one assay’ paradigm, it is impractical to run a different test for each drug, and most pathology laboratories currently use only one platform. Hence, it is important to verify the interchangeability of these assays.

The intratumoral heterogeneity in PD-L1 expression is also important to consider [16]. PD-L1 testing is mainly conducted on biopsy specimens, which may not be representative of the whole tumor. This may lead to false positive or negative results, particularly for small tissue specimens [17]. In cases of false negative results, this could lead to under-treatment of the patients. In turn, this could explain why all biomarker assessments of the 4 clinical trial antibody clones have reported a small fraction of patients with PD-L1-negative tumors who responded to anti PD-1/PD-L1 agents [16, 18].

In the present study, we aimed to 1) compare the analytic results between 4 different PD-L1 IHC and scoring systems, and 2) evaluate the correlation of PD-L1 expression between tissue microarray (TMA) specimens and the corresponding resected specimen to better understand the frequency of discrepancies and the underlying characteristics.

## RESULTS

### PD-L1 expression in tumor cells from whole-tissue sections (WTS)

**Figure 1:**
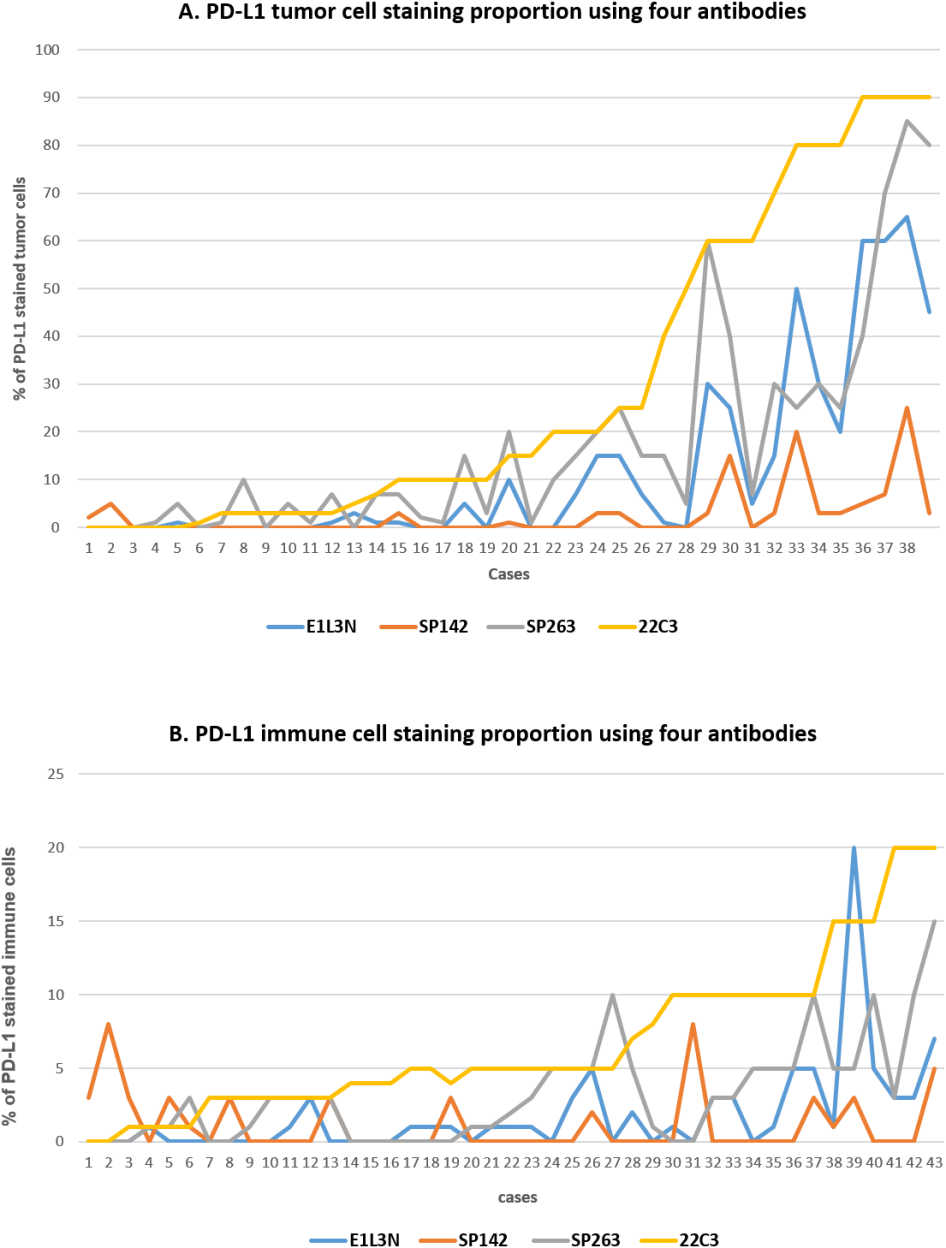
Proportions of staining of PD-L1 in tumor cells **(A)** and immune cells **(B)** for each case and assay among the 38 PD-L1-positive cases. (A) The 22C3 assay showed the highest tumor proportion score (TPS), whereas the SP142 assay showed the lowest TPS. The SP263 and E1L3N assays showed similar TPSs. (B) The positive rate was significantly lower in the immune cells than in the tumor cells.

**Figure 2:**
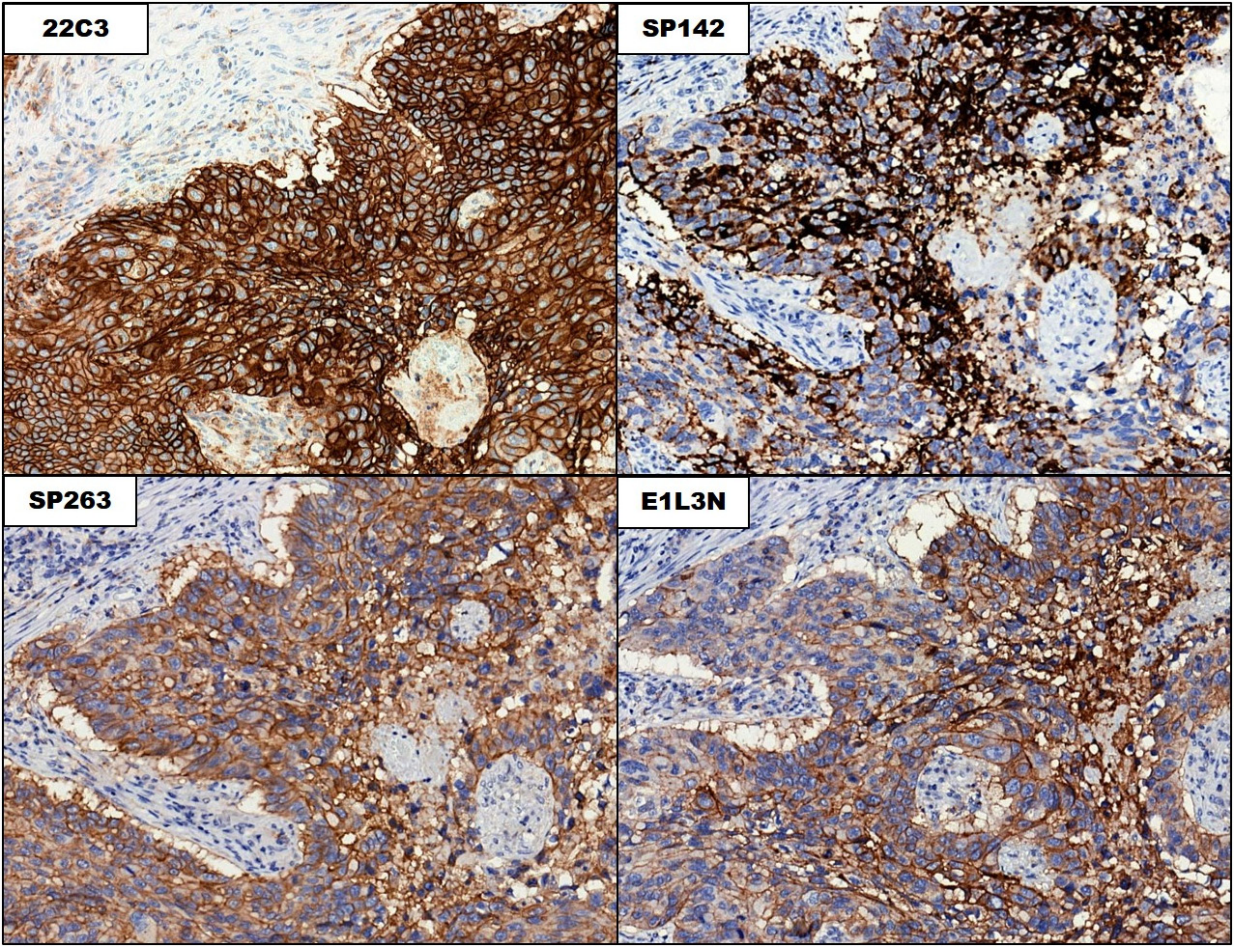
Staining patterns in the tumor cells in the 4 PD-L1 immunohistochemical assays The Figure shows matched regions on consecutive slides stained with the indicated assays (20× magnification).

In the training set, PD-L1 expression in the tumor cells was observed in 40.0% (20/50), 38.0% (19/50), 18.0% (9/50), and 30.0% (15/50) of cases in the 22C3, SP263, SP142, and E1L3N assays, respectively. In the validation set, 30.0% (14/47), 30.0% (14/47), 14.9% (7/49), and 17.0% (8/47) of cases showed a tumor proportion score (TPS) >1% in the 22C3, SP263, SP142, and E1L3N assays, respectively. There was no difference in tumor cell PD-L1 expression between the sets. Among the total 97 cases, 38 showed PD-L1 tumor cell positivity in at least 1 assay; 22C3 showed the highest TPS, followed by SP263 and E1L3N, whereas SP142 showed the lowest TPS (Figure 1A). The 22C3 assay displayed the strongest membranous and cytoplasmic staining (Figure 2). SP142 showed strong intensity, but punctate and discontinuous membranous staining, reflecting the amplification component used in the detection system for this assay. SP263 and E1L3N showed similar staining intensities. In all 4 assays, the PD-L1 expression in the tumor cells showed a heterogeneous pattern.

### PD-L1 expression in immune cells from WTS

In the training set, PD-L1 expression in immune cells was observed in 36% (18/50), 28% (14/50), 12% (6/50), and 18% (9/50) of cases in the 22C3, SP263, SP142, and E1L3N assays, respectively. In the validation set, PD-L1 expression was observed in 40% (19/47), 34% (16/47), 17% (8/47), and 32% (15/47) of cases, respectively. In all assays, the proportion of positive immune cells was significantly lower than that of the tumor cells (Figure 1B). Furthermore, the rate of immune cell positivity varied more than that in the tumor cells. PD-L1 co-expression in both tumor and immune cells was observed in 32% (31/97), 23% (22/97), 10% (10/97), and 11% (11/97) of cases in the 22C3, SP263, SP142, and E1L3N assays, respectively.

### Comparison of PD-L1 status between the 4 assays

Next, the PD-L1 status for the 97 NSCLC cases was compared after classifying the cases according to the combined pre-specified and selected cut-offs (Table 1 and Table 2). The 22C3 and SP263 assays displayed high concordance rates, with κ values of >0.7 for cut-offs of 1–25%; however, the positive rate was 3 times higher with the 22C3 assay for the 50% cut-off (κ value=0.467, moderate agreement; Table 2). This may be due to the difference in the TPS between the assays (Figure 1A). The SP142 assay showed the lowest positive rate for all cut-offs, even after considering the positive rate of the immune cells. The E1L3N assay showed a positive rate between that of the 22C3/SP263 and SP142 assays for cut-offs of 1–25%, and a similar rate to the SP263 assay for a cut-off of 50%.

**Table 1:** Comparison of PD-L1 status between four assays using various cut-offs

assays/cut-offs	Numbers of positive cases (%)				
	≥1%	≥5%	≥10%	≥25%	≥50%
**22C3**	34/97 (35%)	28/97 (29%)	26/97 (27%)	15/97 (15%)	12/97 (12%)
**SP263**	33/97 (34%)	26/97 (27%)	19/97 (19%)	11/97 (11%)	4/97 (4%)
**SP142**	16/97 (16%)	6/97 (6%)	3/97 (3%)	1/97 (1%)	0
**E1L3N**	23/97 (24%)	17/97 (18%)	13/97 (13%)	8/97 (8%)	4/97 (4%)

Table 2Pairwise comparison of PD-L1 tumor cells (upper) and concordance (lower) of the four PD-L1 assays

**Table:** 

**22C3**																										
**SP263**		**0-1**	**1-5**	**5-10**	**10-25**	**25-50**	**>50**	**total**	**SP142**		**0-1**	**1-5**	**5-10**	**10-25**	**25-50**	**>50**	**total**	**E1L3N**		**0-1**	**1-5**	**5-10**	**10-25**	**25-50**	**>50**	**total**
	**0-1**	73	2	0	0	0	0	**75**		**0-1**	63	5	4	6	2	2	**82**		**0-1**	63	4	3	1	1	1	**75**
	**1-5**	6	1	0	0	0	0	**7**		**1-5**	2	0	0	2	2	4	**10**		**1-5**	2	1	1	3	0	0	**7**
	**5-10**	3	0	0	0	0	0	**3**		**5-10**	0	0	0	0	0	2	**2**		**5-10**	0	0	0	1	1	1	**3**
	**10-25**	0	4	0	0	0	0	**4**		**10-25**	0	0	0	0	0	2	**2**		**10-25**	0	0	0	1	2	1	**4**
	**25-50**	0	3	0	1	0	0	**4**		**25-50**	0	0	0	0	0	1	**1**		**25-50**	0	0	0	0	0	4	**4**
	**≥50**	0	0	2	1	1	0	**4**		**≥50**	0	0	0	0	0	0	**0**		**≥50**	0	0	0	0	0	4	**4**
	**total**	**82**	**10**	**2**	**2**	**1**	**0**	**97**		**total**	**65**	**5**	**4**	**8**	**4**	**11**	**97**		**total**	**65**	**5**	**4**	**8**	**4**	**11**	**97**
									**SP263**																	
									**SP142**		**0-1**	**1-5**	**5-10**	**10-25**	**25-50**	**>50**	**total**	**E1L3N**		**0-1**	**1-5**	**5-10**	**10-25**	**25-50**	**>50**	**total**
										**0-1**	70	3	4	5	0	0	**82**		**0-1**	71	2	1	1	0	0	**75**
										**1-5**	2	0	1	2	3	2	**10**		**1-5**	1	1	3	2	0	0	**7**
										**5-10**	0	0	0	0	1	1	**2**		**5-10**	0	0	1	2	0	0	**3**
										**10-25**	0	0	0	0	2	0	**2**		**10-25**	0	0	0	2	2	0	**4**
										**25-50**	0	0	0	0	0	1	**1**		**25-50**	0	0	0	0	2	2	**4**
										**≥50**	0	0	0	0	0	0	**0**		**≥50**	0	0	0	0	2	2	**4**
										**total**	**72**	**3**	**5**	**7**	**6**	**4**	**97**		**total**	**72**	**3**	**5**	**7**	**6**	**4**	**97**
																		**E1L3N**								
																		**SP142**		**0-1**	**1-5**	**5-10**	**10-25**	**25-50**	**>50**	**total**
																			**0-1**	73	2	0	0	0	0	**75**
																			**1-5**	6	1	0	0	0	0	**7**
																			**5-10**	3	0	0	0	0	0	**3**
																			**10-25**	0	4	0	0	0	0	**4**
																			**25-50**	0	3	0	1	0	0	**4**
																			**≥50**	0	0	2	1	1	0	**4**
																			**total**	**82**	**10**	**2**	**2**	**1**	**0**	**97**

**Table:** 

22C3																				
		**1%**	**5%**	**10%**	**25%**	**50%**			**1%**	**5%**	**10%**	**25%**	**50%**			**1%**	**5%**	**10%**	**25%**	**50%**
**SP263**	**1%**	0.863	0.785	0.782	0.524	0.43	**SP142**	**1%**	0.468	0.54	0.581	0.655	0.667	**E1L3N**	**1%**	0.662	0.761	0.7	0.676	0.556
	**5%**	0.74	0.744	0.737	0.666	0.556		**5%**	0.155	0.214	0.236	0.425	0.516		**5%**	0.547	0.687	0.734	0.776	0.718
	**10%**	0.603	0.695	0.741	0.716	0.582		**10%**	0.107	0.146	0.16	0.297	0.369		**10%**	0.43	0.552	0.594	0.75	0.77
	**25%**	0.369	0.479	0.518	0.823	0.852		**25%**	0.036	0.05	0.055	0.108	0.137		**25%**	0.275	0.363	0.394	0.659	0.778
	**50%**	0.142	0.192	0.21	0.381	0.467									**50%**	0.142	0.192	0.21	0.381	0.467
							**SP263**													
									**1%**	**5%**	**10%**	**25%**	**50%**			**1%**	**5%**	**10%**	**25%**	**50%**
							**SP142**	**1%**	0.501	0.641	0.756	0.786	0.358	**E1L3N**	**1%**	0.703	0.864	0.758	0.583	0.243
								**5%**	0.169	0.236	0.338	0.552	0.369		**5%**	0.584	0.734	0.864	0.751	0.337
								**10%**	0.117	0.16	0.232	0.399	0.26		**10%**	0.462	0.594	0.777	0.905	0.435
								**25%**	0.04	0.055	0.082	0.151	0.39		**25%**	0.297	0.394	0.539	0.825	0.637
															**50%**	0.154	0.21	0.3	0.503	0.478
														**E1L3N**						
																**1%**	**5%**	**10%**	**25%**	**50%**
														**SP142**	**1%**	0.65	0.744	0.879	0.625	0.358
															**5%**	0.274	0.378	0.483	0.693	0.79
															**10%**	0.186	0.261	0.342	0.524	0.556
															**25%**	0.065	0.093	0.126	0.208	0.39

The concordance of the PD-L1 positive rate improved with the assay-specific cut-off. Table 1 shows the κ values of the 4 assays in pairs for each cut-off. The highest κ values in each table were observed when assay-specific criteria were applied. In the E1L3N assay, the 10% cut-off showed the highest agreement with the commercial assay. The 10% and 25% cut-offs showed the highest agreements for the E1L3N and SP263 assays, respectively (κ value=0.905, very good agreement; Table 2).

We compared PD-L1 status which means above or below the specific cut-off threshold for each assay. Ten of the 97 cases (10%) were above the cut-offs used for all 4 assays, indicating that PD-L1 positivity would be concordant regardless of the assay used. Moreover, 79 of 97 (81%) cases were found to be below the cut-off value for PD-L1 negativity, regardless of the assay used. Eight cases (8%) showed variations in the classification, being classified either above or below the assay-specific cut-off. These data indicate that using an alternative validated assay and an assay-specific scoring algorithm to evaluate PD-L1 expression would yield different results in only approximately 8% of cases. However, the replacement of the validated cut-off for each assay with any other cut-off reduces the overall agreement in comparison with the reference standard.

### Correlation of PD-L1 expression between TMA sections and the corresponding WTS

**Figure 3:**
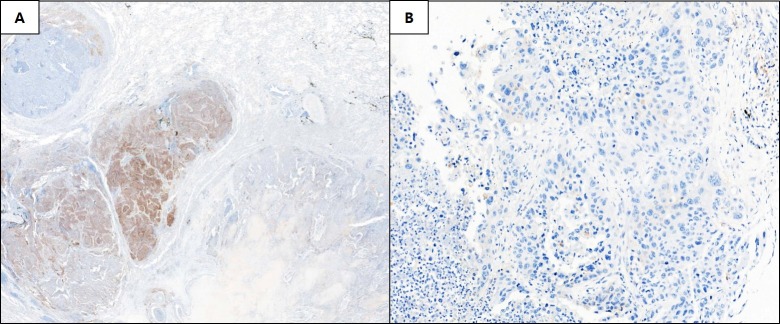
Representative images of discordant non-small cell lung cancer cases between the whole-tissue section (A, 10× magnification) and matched tissue microarray specimen (B, 20× magnification)

We used the 22C3 assay for assessing the TMA sections, and obtained PD-L1 IHC results for matched WTS and TMA specimens in 50 cases in the training set. With regard to the TPS, among the 50 cases, 29 (58%) cases were PD-L1 negative (TPS=0) and 14 (28%) were PD-L1-positive (TPS≥1%) in both the WTS and TMA specimens. Seven of 50 (14%) cases exhibited discordant PD-L1 expression, with negative findings in the TMA compared with those in the WTS (TPS=1–50%) (Figure 3). Among the 14 cases showing PD-L1 expression in both the TMA and WTS specimens, one case showed a significant difference in the positive rate (90% vs. 15%). When the 50% cut-off was applied, the PD-L1 status was interpreted differently.

For immune cells, among the 50 cases, 18 (36%) cases were PD-L1-negative and 11 (22%) were PD-L1-positive in both the WTS and TMA specimens. Although 21 of 50 (42%) cases showed discordant PD-L1 expression in the immune cells between the WTS and TMA, the immune cell-positive rate was low, ranging between 1–15%.

## DISCUSSION

In the present study, we demonstrated that 1) the PD-L1 status could be designated differently depending on the assay-specific cut-off, and 2) small biopsy/TMA specimens underestimated the PD-L1 status, as compared with resected specimens.

Our results showed that the 22C3 and SP263 assays were similar in terms of the PD-L1 staining performance. The E1L3N assay, an LDT, exhibited a similar staining pattern, although the TPS was slightly lower than that of the above-mentioned 2 assays. The SP142 assay generally stained fewer tumor cells, consistent with the Blueprint Project Phase I results [19]. All assays labelled immune cells, although there was less precision than that in tumor cell labelling. This may be because the pathologists did not predetermine how they would define/evaluate immune cell staining [19]. Herein, we performed 4 different assays on specimens divided into training and validation sets according to the paraffin block age, and confirmed that there was no difference in the PD-L1 positive rate between the groups, indicating that the age of the paraffin block did not affect the PD-L1 staining.

A negative or positive PD-L1 status depends on the cut-off used and is crucial for subsequent treatment decisions. When various cut-offs, including assay-specific criteria, were used to designate the PD-L1 status in the present study, the 22C3 and SP263 assays displayed similar positive rates in the overall range, followed by the E1L3N assay, whereas the SP142 assay showed the lowest rate. Because of differences in the TPS for each assay, the application of the same cut-off to all assays resulted in a large positive rate discordance. When the assay-specific cut-offs were applied, the overall agreement rate was high, and only approximately 8% of cases exhibited variable PD-L1 status. Especially, only a few cases designated as PD-L1-negative showed values below the cut-off in the 22C3 or SP263 assays but were PD-L1-positive with the SP142 assay, owing to variations in the assay-specific cut-off. Therefore, it may be possible to infer the PD-L1 staining level results between assays, while using clinically defined assay-specific cut-offs for each drug.

Further, we performed laboratory-developed PD-L1 tests using the E1L3N clone. The current companion/complimentary PD-L1 IHC assay was developed using a specific clinical program covering the staining systems and scoring/interpretation guidelines for a specific drug, and validated using the patient's clinical outcome. However, several LDT antibodies and IHC protocols have been validated using tissue samples. Herein, we compared the staining performance of these LDT antibodies with that of commercial assays. As a result, we found that LDTs are not inferior to commercial assays and can be used interchangeably when using the appropriate cut-offs. Thus, following strict validation of the analysis and clinical response, LDTs may be used as a screening method to validate the results of commercial assays.

PD-L1 staining concordance between biopsy and resected specimens is important. In a recent study, significant discordance of PD-L1 expression using the E1L3N and SP142 clones between the TMA core and the corresponding WTS in 49 NSCLC cases was observed [16]. Similarly, a comparison of PD-L1 expression using the SP142 clone between preoperative biopsy specimens and corresponding resected specimens in 160 NSCLC patients also found significant discordance (overall discordance rate=48%; κ value=0.218) [17]. In the present study, we used the 22C3 assay for comparison, as 22C3 showed the highest TPS and may reduce the intratumoral heterogeneity in PD-L1 expression. Despite the low rate of discordance compared with other studies, the PD-L1 expression in small lung biopsy specimens can be misleading; hence, multiple biopsies from different areas of the tumor may be needed to validate the IHC evaluation results [17].

To our knowledge, this is the first comparative study of PD-L1 IHC assays, including LDT assays, in a NSCLC resection cohort. However, the lack of clinical response data for anti–PD-1/PD-L1 therapy is an important limitation of our analysis. Because the ultimate goal of determining PD-L1 IHC status is to predict the therapeutic response, the debate regarding the use of PD-L1 IHC assays may be settled using relevant clinical trial data for the drug being considered. Moreover, the threshold of the drug, rather than the assay, is a key parameter. Nevertheless, further studies are needed to determine the most appropriate cut-off and compare the sensitivity of the assays according to the therapeutic response.

In conclusion, despite the different PD-L1 assay staining results, the PD-L1 status observed with assay-specific cut-offs was similar, suggesting that appropriate clinically defined algorithms or cut-offs should be applied to each assay. Furthermore, to consider LDTs as equivalent to the commercially available companion/complementary assays, rigorous validation is required. Hence, it is important to standardize the PD-L1 assays and enhance communications between clinicians, pathologists, and providers to establish both national and international guidelines for PD-L1 testing.

## MATERIALS AND METHODS

### Study design and case selection

We retrospectively collected formalin-fixed paraffin-embedded whole-tissue sections (WTS) from 97 resected NSCLC cases at Seoul National University Bundang Hospital. Fifty NSCLC WTS (32 adenocarcinomas and 18 squamous cell carcinomas) resected from 2010 to 2011 were assigned as the ‘training set’, and 47 NSCLC WTS (32 adenocarcinomas, 14 squamous cell carcinomas, and 1 pleomorphic carcinoma) resected from 2015 to 2016 were assigned as the ‘validation set’. For the training set samples, a TMA was constructed using 2-mm-diameter cores derived from the representative tumor areas of formalin-fixed paraffin-embedded tissue blocks by SuperBioChips Laboratories (Seoul, Korea), as previously described [20]. This study was approved by the local ethics committee.

### Immunohistochemical analysis

Ninety-seven WTS slides were stained using the Dako (Carpinteria, CA, USA.) and Ventana (Tucson, AZ, USA) platforms and their PD-L1 IHC assays. The Dako pharmDx assay was stained with an anti-PD-L1 22C3 mouse monoclonal primary antibody with the EnVision FLEX visualization system on a Dako Autostainer Link 48 system, along with negative control reagents and cell line run controls, as per the manufacturer's instructions [5]. For the Ventana assay, the sections were stained with an anti-PD-L1 (SP263) rabbit monoclonal primary antibody using the OptiView DAB IHC Detection kit on the BenchMark XT automated staining platform [9]. For the SP142 assay, the sections were stained with an anti-PD-L1 (SP142) rabbit monoclonal primary antibody using the OptiView DAB IHC Detection kit, followed by the OptiView Amplification Kit, on the BenchMark XT automated platform [6]. For the E1L3N assay, the sections were stained with an anti-PD-L1 (E1L3N) rabbit XP monoclonal primary antibody (1:100; Cell Signaling Technology, Danvers, MA, USA) with the OptiView DAB IHC Detection kit on the BenchMark XT automated platform.

### Evaluation of PD-L1 expression

Two pathologists (HK and JHC) scored the 388 WTS IHC slides (97 cases × 4 assays) and 50 TMA IHC cores independently, and recorded 438 raw percentages of tumor cells and immune cells expressing PD-L1.

PD-L1 expression was defined in tumor cells if membranous alone or membranous and cytoplasmic staining was present [3, 4, 6, 7, 9]. The PD-L1 scoring in tumor cells was expressed as a percentage of the stained cells in the entire tumor section, and was termed the tumor proportion score (TPS) [3, 7]. The TPS was estimated in increments of 5%. PD-L1-positive intratumoral and peritumoral immune cells located at the interface between the tumor and non-neoplastic lung were also scored. For lymphocytes, membranous and cytoplasmic staining could not be reliably distinguished because of the small size. The PD-L1 scoring in immune cells was expressed as a percentage of the stained immune cells in the tumor area, and estimated in 5% increments [6, 8]. The staining intensity was not included in the evaluation. For TMA, sections with ≥100 viable carcinoma cells were evaluated.

### PD-L1 scoring and analysis

We evaluated the PD-L1 expression using a scoring system with various cut-offs (1%, 5%, 10%, 25%, and 50%), including assay-specific cut-offs for the 22C3, SP263, and SP142 assays. The TPS was a common element of PD-L1 scoring in all assays. For 1 assay (SP142), the area of PD-L1-positive immune cells was integrated into the scoring system [6, 8].

### Statistical analysis

Statistical analysis was conducted using SPSS (version 21.0; SPSS, Inc., Chicago, IL), with Cohen's κ coefficient of agreement performed for comparing the dichotomized expression values between 2 assays.
